# Characterization and mechanism of seed dormancy in *Symplocos paniculata*

**DOI:** 10.3389/fpls.2023.1322238

**Published:** 2024-01-08

**Authors:** Qiaoyu Tang, Yunzhu Chen, Lijuan Jiang, Jingzhen Chen, Changzhu Li, Wenbin Zeng, Qiang Liu, Peiwang Li

**Affiliations:** ^1^ College of Life Science and Technology, Central South University of Forestry and Technology, Changsha, China; ^2^ State Key Laboratory of Utilization of Woody Oil Resource, Hunan Academy of Forestry, Changsha, China

**Keywords:** *Symplocos paniculate*, seed dormancy, endogenous inhibitor, fatty acids, phenolics

## Abstract

*Symplocos paniculata* is a highly desirable oil species for biodiesel and premium edible oil feedstock. While germplasm preservation and breeding are crucial, the severity of seed dormancy poses a challenge to successful germination. We employed *S. paniculata* seeds as experimental materials and conducted an investigation into the types and causes of seed dormancy by analyzing the morphology and developmental characteristics of its embryo, exploring the water permeability property of the endocarp, and examining the presence of endogenous inhibitors, aiming to establish a theoretical foundation for overcoming seed dormancy and maximizing germplasm resource utilization. The findings revealed that the seed embryo had matured into a fully developed embryo, and no dormancy in terms of embryo morphology was observed. Upon reaching maturity, the endocarp of seeds undergoes significant lignification, resulting in notable differences in water absorption between cracked and intact seeds. The impermeability of the endocarp is one of the factors contributing to mechanical restriction. The different phases of endosperm extraction exerted varying effects on the germination of Chinese cabbage seeds, with the methanol phase exhibiting the most potent inhibitory effect. The presence of endogenous inhibitors emerged as the primary factor contributing to physiological dormancy in seeds. GC-MS analysis and validation trials revealed that fatty acids and phenolics, including hexadecanoic acid, oxadecanoic acid, and m-cresol, constituted the main types of endogenous inhibitory compounds found within the endosperm. These findings suggest that the seed dormancy in *S. paniculata* seeds has endocarp mechanical restriction, and the presence of endogenous inhibitors causes physiological dormancy.

## 1 Introduction

*Symplocos paniculata* is an indigenous deciduous shrub in China, boasting both ecological and economic significance. This remarkable species is also renowned for its fireproof properties, earning it the classification of a national second-class fireproof tree species. Moreover, its roots and leaves possess valuable medicinal properties (Ruchi et al., 2011). With its multifaceted applications and immense developmental potential, this versatile tree offers a plethora of opportunities. Notably, every part of the fruit contains oil with considerable content reaching up to an impressive 36.6%. The predominant oils and fats found in *S. paniculata* oil consist of hexadecanoic acid, oxadecanoic acid, oleic acid, linoleic acid, and linolenic acid, boasting a remarkable mass fraction of unsaturated fatty acids reaching an impressive 76.74% or higher (Li et al., 2023). The oil obtained after refining exhibits no significant deviation in terms of color, transparency, acid value, soap value, odor, and other characteristics when compared to ordinary cooking oil. Moreover, this peanut-like oil not only contributes to the health benefits associated with edible vegetable oils but also serves as a vital component among China’s primary raw materials for edible vegetable oils (Na et al., 2006; Semwal et al., 2011). The oil of *S. paniculata* possesses a semi-dry nature, with its fatty acid composition and quality score meeting the standards for biodiesel production. Moreover, it can serve as an exceptional raw material of superior quality for processing and generating biodiesel (Liu et al., 2015; Liu Q. et al., 2016). The versatile application of this oil extends beyond its use as a culinary delight and biofuel, encompassing an array of industrial processes. It serves as a lubricating agent in the machinery industry, imparts softness to wool and eliminates static electricity during textile production, acts as a blending catalyst in printing ink manufacturing, and even finds utility in soap production within the chemical sector (Na et al., 2006; Li et al., 2023).

However, *S. paniculata* seeds exhibit biennial germination characteristics (Ruchi et al., 2011), characterized by a profound dormancy phenomenon. In natural conditions, two winters and a summer are required for them to emerge as seedlings. A significant proportion of seeds lose vitality during the lengthy process of dormancy and germination, thereby hindering the controllability of *S. paniculata* sexual reproduction breeding and severely impeding the selection process and application of this exceptional germplasm resource. Therefore, to enhance the development and utilization of its germplasm resources, it is imperative to identify the type and underlying cause of seed dormancy and establish a theoretical foundation for subsequent dormancy alleviation. Seed dormancy, a form of resistance acquired through the long-term process of seed adaptation, prevents mature viable seeds from immediately germinating in favorable environmental conditions, necessitating a relatively static stage known as seed dormancy (Fu et al., 2013; Seung et al., 2018; Nagel et al., 2019; Lennon et al., 2021). Dormancy can arise from various factors, such as embryonic characteristics (e.g., underdevelopment and/or physiological inhibitory mechanisms) or the seed/fruit coat (Finch-Savage and Leubner-Metzger, 2006; Zhang et al., 2019). There are five distinct classes of dormancy: physiological dormancy (PD), morphological dormancy (MD), morphophysiological dormancy (MPD), physical dormancy (PY), and combinational dormancy (PY + PD) (Baskin and Baskin, 2014). The determination of the specific type of dormancy is crucial for devising appropriate strategies to break it.

Extensive research has been conducted on various mechanisms of seed dormancy, including the characterization of seed dormancy in *Rhamnus ussuriens*is (Liu et al., 2020), which unveiled a comprehensive dormant type resulting from the synergistic effect of a seed coat barrier and endogenous inhibitory substances, thus inducing dormancy. Furthermore, an investigation into the characterization of dormancy in *Iris scariosa* revealed that the primary causes are attributed to binding between the seed coat and endosperm (Zhang et al., 2016). The primary cause of seed dormancy in *Amsonia elliptica* is attributed to the limited growth potential of the embryonic seed (i.e., physiological dormancy) (Lee et al., 2022). Furthermore, it should be noted that both the type and reason for seed dormancy vary among different species. For instance, while endogenous inhibitors present in the endosperm of *S. paniculata* seeds have been identified as a contributing factor to their dormancy, further research is required to determine the specific types and concentrations of these inhibitors (Liu L. et al., 2016). However, to the best of our knowledge, there is currently no available information regarding the precise dormancy class of *S. paniculata*. Therefore, our study aimed to analyze the embryo’s morphology and developmental characteristics, assess the endocarp’s water permeability, and investigate the presence of endogenous inhibitors in order to elucidate both the type and mechanism of seed dormancy. Our findings provide valuable insights for establishing a theoretical foundation towards overcoming seed dormancy and maximizing germplasm resource utilization of *S. paniculata*.

## 2 Materials and methods

### 2.1 Acquisition of experimental materials

The seeds of *S. paniculata* were collected biweekly from the experimental station at the Hunan Academy of Forestry, located in Changsha city, China (113.00°E, 28.11°N), during the fruit development period in 2022 spanning from 30 to 190 Days after flowering (DAF). The collected seeds were divided into three groups for different experiments. One group was preserved in FAA fixative (a mixture of formaldehyde, acetic acid, and ethyl alcohol, volume ratio 5:5:90) and used to create paraffin sections for observing embryo morphology. Another group was kept fresh at 4°C and used for analyzing seed properties such as size, mass, and viability. The third group was stored at -80°C for endogenous inhibitor analysis. The mature *S. paniculata* seeds utilized in this study exhibit deep dormancy, rendering them non-germinable during the first year, and only demonstrate a 30% germination rate in the second year. The commercial Chinese cabbage (*Brassica rapa* var. Glabra) seeds were purchased, exhibiting a purity level of ≥96.0% and a germination rate of ≥85.0%. The Chinese cabbage seeds are commonly employed in seed dormancy inhibitor validation experiments due to their non-dormant nature, high germination rate, easy germination process, and short germination cycle.

### 2.2 Methods

#### 2.2.1 Seed properties analysis

The horizontal and vertical diameters of the *S. paniculata* seeds (20 grains) were measured using a vernier caliper with a precision of 0.01mm. while the 1000-seed mass was determined using an electronic balance with a sensitivity of 0.001 g. The viability of fresh *S. paniculata* seeds was assessed by staining them with 1% TTC (2,3,5-triphenyltetrazolium chloride) in the dark at 25°C for 24 hours. Seed viability was confirmed using stereo-microscopy (SZX2-ILLT; Olympus Corporation, Tokyo, Japan), and seeds were considered viable when both the embryo and endosperm exhibited a red stain. Stereo-microscopy was employed to observe the external morphology and internal structural characteristics of the seeds.

#### 2.2.2 The observation of endocarp lignification and the determination of water absorption rate

The seeds of *S. paniculata* at different developmental stages were transversely cut from the middle, and the sections were immersed in a solution containing phloroglucinol (0.5%) and hydrochloric acid (6 mol/L) for a duration of 3-5 minutes. The endocarp exhibited a red lignification pattern. The degree of lignification in *S. paniculata* seed endocarps was examined using a stereo-microscope. A total of 100 mature *S. paniculata* seeds, including both punctured (dissecting needles were used to puncture the endocarp to the endosperm, creating three holes in each seed) and intact seeds, were individually placed in a 200 mL beaker and submerged with water to cover the entire seed surface. Subsequently, the seeds were incubated at 25°C for 78 hours, with periodic removal and weighing after draining the water from the seed surface until no further change in mass was observed.

#### 2.2.3 Microscopic examination of embryonic morphogenesis

The fruits of *S. paniculata* were immediately fixed in FAA fixative after harvesting at different developmental stages, followed by dehydration in a gradient ethanol solution (70%, 85%, 95%, and 100%), and embedding in wax. The embedded wax blocks were sectioned into 12*μ*m thick wax strips and placed onto slides, followed by unfolding the slices on a drying machine set at 37°C. Subsequently, the sections were subjected to drying and deparaffinization processes, stained with hematoxylin and eosin, and finally fixed using neutral gum. Finally, the sections were examined under an electron microscope (Li S. J. et al., 2022).

#### 2.2.4 Extraction and analysis of endosperm endogenous inhibitors from *S. paniculata* seeds

Two grams of *S. paniculata* endosperm powder (including embryo) was placed in a 50 mL centrifuge tube and subjected to liquid nitrogen refrigeration. Then, 40 mL of 100% methanol solution was added, followed by ultrasonic extraction at 40 KHZ and 40°C for one hour. The mixture was allowed to stand overnight at 4°C before filtering. The residual leaching solution was subjected to an additional round of leaching by adding 40 mL of 100% methanol solution, which was repeated three times. Subsequently, all the leaching solutions were combined and concentrated under reduced pressure steam at 40°C with methanol, resulting in a final volume of 50 mL after dilution with distilled water. The method of solvent separation in the system involved preliminary separation of methanol extracts from each part of the seed, followed by extraction with petroleum ether, ethyl ether, ethyl acetate, methanol phase and water phase separately. The resulting extraction liquids were then enriched through evaporation using a rotary evaporation apparatus and transferred into 20 mL capacity containers before being stored in a refrigerator at 4°C.

#### 2.2.5 Analysis of germination inhibition in the different endosperm extraction phase

In this study, Chinese cabbage seeds were utilized as the primary material to compare the germination experiment results of seed endosperm solutions extracted using different solvents. The Petri dish, covered with a layer of filter paper, was individually supplemented with 5mL of distinct extracts. Following the evaporation of organic solvents, 5mL of distilled water was added to each dish, while an equal volume of distilled water was added to the control group. Subsequently, the petri dish was placed in a constant temperature and light incubator set at 25°C for germination experimentation. The germination process was monitored by measuring the extension of the radicle to 1cm, with counts taken every 8 hours. Germination was considered complete when the number of germinated seeds did not exceed 1% for three consecutive days. The final measurements included seedling height and root length for each treatment group.

#### 2.2.6 Identification of methanol phase in endosperm using GC-MS analysis

A 10 ml portion of methanol phase was used to concentrate the endosperm, and the condensed matter resulting from boiling away the solvent on a pan was then further concentrated to 2 ml. The solute in the methanol phase was subsequently analyzed using Gas Chromatography-Mass Spectrometer (GC-MS). The determination conditions for GC-MS analysis were as follows: A DB-5MS capillary column (30 m × 250μmi.d., 0.25μm film thickness, Agilent J & W Scientific, Folsom, CA, USA) was employed for gas chromatography to separate the derivatized material. Helium gas at a constant flow rate of 1 mL/min was used as the carrier gas. An autosampler injected a sample volume of 1 µL with a split ratio of 1:10. The injection port temperature was set at 280°C, while the transmission line and ion source temperatures were maintained at 320°C and 230°C, respectively. The warming program initiated with an initial temperature of 50°C for a duration of 0.5 min, followed by a gradual increase to reach 320°C at a rate of 15°C/min, which was then sustained for a period of 9 min. The mass spectrometry analysis was conducted using the full-scan method at a scan rate of 10 spec/s, an Electron Energy of -70 V, and a solvent delay of 3 min (Fiehn et al., 2008).

#### 2.2.7 Impact of key inhibitors on the germination and growth of Chinese cabbage seeds

According to the GC-MS appraisal results, we identified the top three metabolites: hexadecanoic acid, octadecanoic acid, and m-cresol. Subsequently, these three commercial standards (Cas No. 57-10-3/57-11-4/108-39-4) were procured from Macklin in China. The hexadecanoic acid, octadecanoic acid, and m-cresol were diluted in methanol solutions to concentrations of 1, 10, 100, and 500 mg/L respectively. Methanol solution was used as a blank control for the germination experiment of Chinese cabbage seeds.

### 2.3 Statistical analysis

The means and standard deviations of the biological replicates (n=3) were calculated using Microsoft Excel 2021. Analysis of variance (ANOVA) was performed to compare germination, seedling height, and root length of Chinese cabbage seeds under different treatments using SPSS 26.0 software. Significant differences were determined using Duncan’s test at a significance level of P<0.05. GraphPad Prism (Version 8.0.2) was utilized for data visualization.

## 3 Results

### 3.1 Seed properties

**Figure 1:**
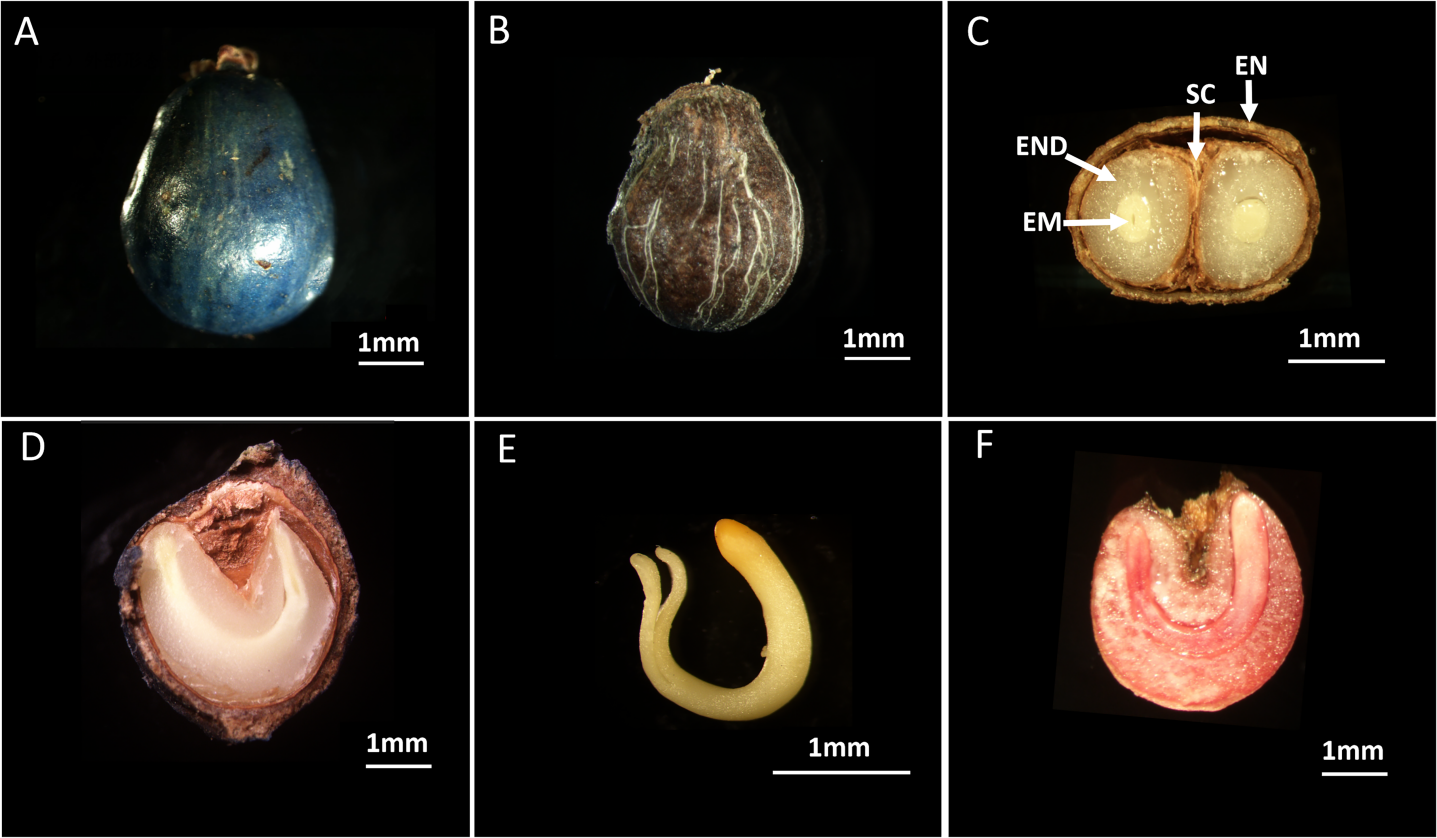
The Morphological characteristics and viability of *S. paniculata.* **(A)** Fruit of *S. paniculate*; **(B)** Seed of *S. paniculata* (with endocarp); **(C)** Horizontal section of seeds; **(D)** Longitudinal section of seeds; **(E)** Embryo of *S. paniculate*; **(F)** Viable seeds show red color under TTC staining. EN, endocarp; SC, seed coat; END, endosperm; EM, embryo.

The *S. paniculata* flowers from the months of April and May, while its fruits are produced during the period from October to November. After the delicate flowers have bloomed, a miraculous transformation takes place - the fertilized ovary evolves into a mesmerizing blue-purple drupe (**Figure 1A**). Simultaneously, the ovule undergoes a metamorphosis and becomes an ovoid seed, boasting dimensions of 4.52 ± 0.59mm for its transverse diameter and 5.85 ± 0.61mm for its longitudinal diameter (**Figure 1B**). Furthermore, this remarkable seed possesses a 1000-seed mass of 47.25 ± 2.82g. The internal examination of the seed structure revealed a composition consisting of four distinct components: the rigid and thick lignified endocarp, the testa, the endosperm, and the embryo (**Figures 1C, D**). Within this kidney-shaped endosperm lies a yellowish-white “U”-shaped embryo with well-developed cotyledons and an elongated hypocotyl (**Figure 1E**). The evaluation of seed viability using tetrazolium solution yielded a remarkable result of 86%, indicating excellent development of the seed embryo (**Figure 1F**).

### 3.2 The seeds of *S. paniculata* possess an endocarp that is richly lignified

**Figure 2:**
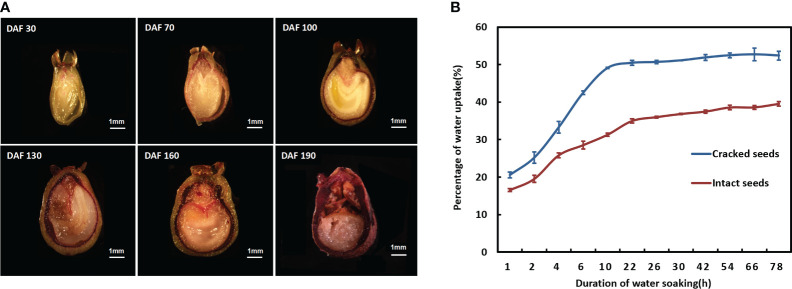
The characterization of endocarp lignification in *S. paniculata.* **(A)** Lignin deposition in the endocarp during various developmental stages; **(B)** Water absorption capacity of intact and cracked seeds. DAF, Days after flowering.

Lignification of the endocarp as a contributing factor to seed dormancy (Gao et al., 2022). The resorcinol staining method was employed to investigate the alterations in lignin deposition within the endocarp of *S. paniculata* seeds during various developmental stages. Subsequently, under a somatic-viewing microscope, the stained endocarp of *S. paniculata* seeds revealed distinct patterns of lignin deposition at different developmental periods, as illustrated in **Figure 2A**. The lignin content of the endocarp progressively increased as the seed developed. During the pre-developmental stage (30-70 DAF), a subtle staining appeared on the endocarp, with a small amount of lignin deposition. In the mid-developmental stage (70-130 DAF), visible lignification occurred in the endocarp, accompanied by an evident enlargement and deepening of the staining site. The endocarp was completely imbued with a deep crimson hue during the ripening stage (130-190 DAF), and this vibrant staining extended throughout its entirety at the mature stage (130-190 DAF).

Further investigation into the water absorption properties of mature seeds exposed to the cracked treatment unveiled that cracked endocarp seeds exhibited a heightened rate of water uptake compared to intact seeds (**Figure 2B**). Within a span of 10 hours, cracked seeds swiftly reached a saturation level of 49.09% before stabilizing. The water absorption of cracked seeds increased significantly to 52.39% after 78 hours, while intact seeds only reached a modest increase of 31.30% after 10 hours and required at least 54 hours to approach saturation levels. The striking contrast implies that the endocarp of *S. paniculata* seeds presents a substantial impediment to water permeability, as its rigid and thick structure severely hampers water absorption, potentially contributing to mechanical dormancy.

### 3.3 The process of embryo development in *S. paniculata* seeds does not involve any morphological maturation

**Figure 3:**
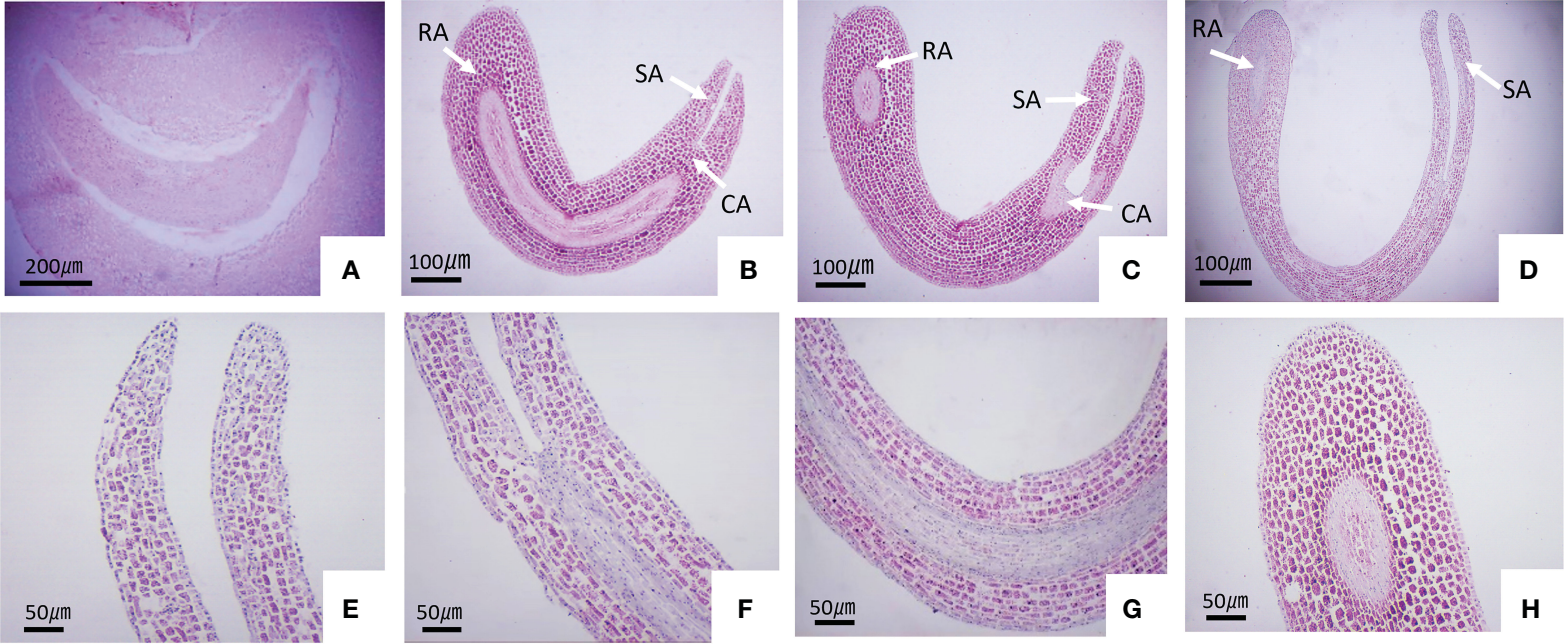
The embryonic development of *S. paniculata* seeds at various stages of growth. **(A)** Pre-torpedo embryo; **(B)** Torpedo embryo; **(C)** Cotyledon embryo; **(D)** Mature embryo; **(E)** Cotyledons in the mature embryo; **(F)** Germ in the mature embryo; **(G)** Hypocotyl in the mature embryo; **(H)** Radicle in the mature embryo. CA, Cotyledon anlage; SA, Meristem onshoot apex; RA, Meristen onroot apex.

To investigate the extent of seed embryo morphology development in *S. paniculata* prior to seed maturity, we conducted a comprehensive analysis of morphological changes throughout the entire developmental process by examining paraffin sections at various stages. The findings revealed that on the 55 DAF, the seed embryo of *S. paniculata* underwent transformation into the pre-torpedo embryo (**Figure 3A**). The meristem of the root tip ceased differentiation once five meticulously arranged and uniform cell layers had been established, while the two cotyledon primordia underwent differentiation towards the upper end morphologically, giving rise to distinct processes. In the epicotyl region, 100 DAF (**Figure 3B**), elongation commenced resulting in torpedo-like embryos. The cotyledon primordia of the torped-shaped embryo underwent further differentiation into symmetrical cotyledons approximately 130 DAF. At the upper morphological end, the shoot end meristem formed the germ, while at the lower morphological end, the root end meristem developed into the radicle. Additionally, the middle meristem between the shoot and root ends transformed into a well-developed hypocotyl. The initial completion of organ differentiation in the young embryo gave rise to a cotyledon-type embryo (**Figure 3C**). Over the course of 175 DAF, as seed development progressed, the cotyledon began to bend and elongate into a mature embryo with well-formed cotyledons, germs, hypocotyls, and radicles (**Figures 3D–H**). Above all, the morphological differentiation of *S. paniculata* seeds is characterized by the completion of the entire embryo structure before maturity, devoid of any signs of ripeness subsequent to embryonic formation. Consequently, it can be inferred that seed dormancy in *S. paniculata* is not attributed to developmental processes associated with embryo form.

### 3.4 The existence of germination inhibitors within the endosperm of *S. paniculata* seeds

#### 3.4.1 Comparison of the inhibitory effects of different extraction phases of endosperm

**Figure 4:**
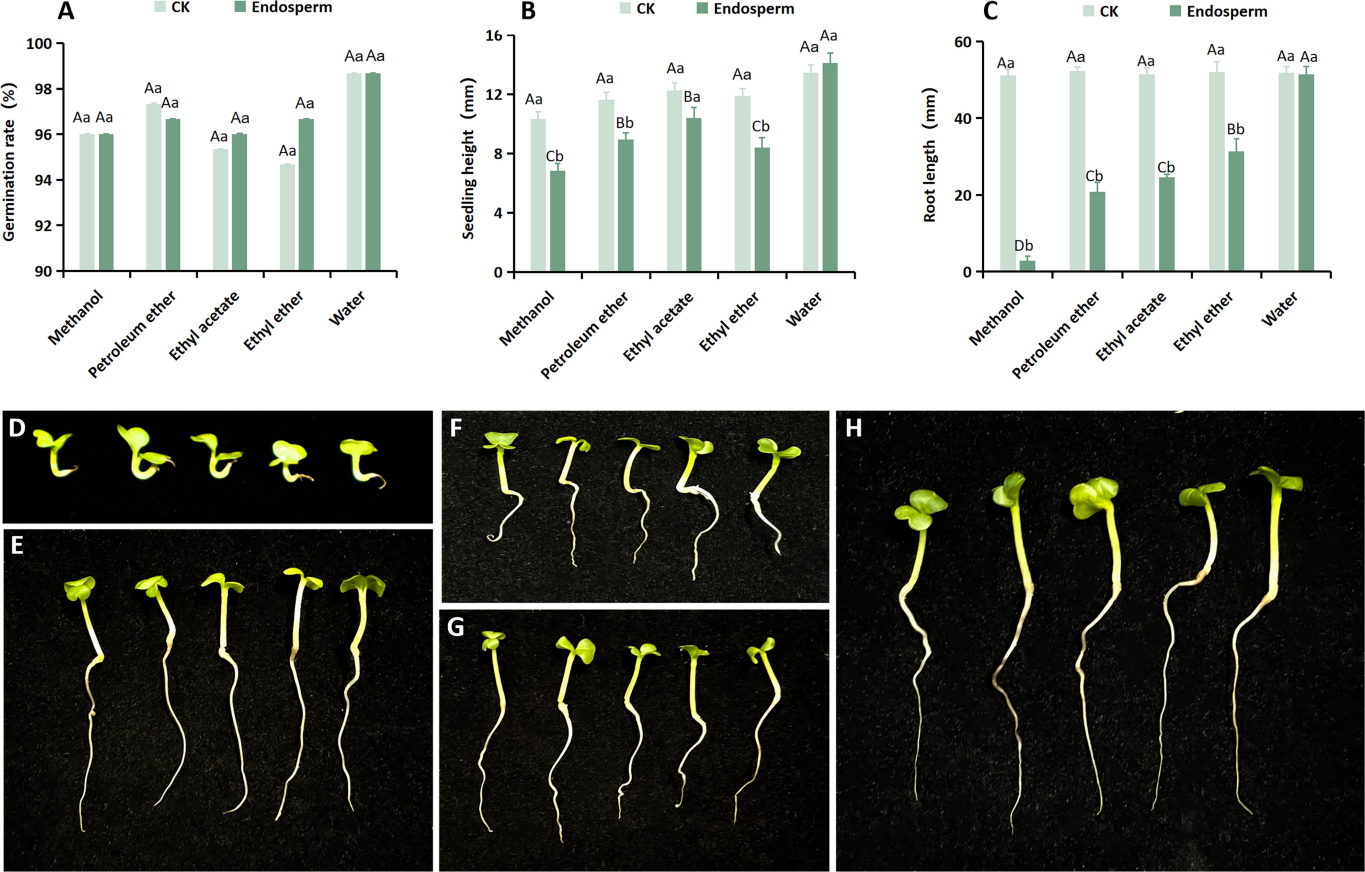
The impact of diverse isolation methods for *S. paniculata* endosperm on the growth of Chinese cabbage. **(A)** Effect of different isolation phases on the germination rate of Chinese cabbage; **(B)** Different isolation phases have different effects on Chinese cabbage seedling height growth; **(C)** Different isolation phases have different effects on Chinese cabbage root length growth; **(D)** Growth of Chinese cabbage under methanol phase treatment; **(E)** Growth of Chinese cabbage under ethyl ether phase treatment; **(F)** Growth of Chinese cabbage under petroleum ether treatment; **(G)** Growth of Chinese cabbage under ethyl acetate phase treatment; **(H)** Growth of Chinese cabbage under water phase treatment. Capital letters indicate differences between separated phases (methanol, petroleum ether, ethyl acetate, ethyl ether and water; P<0.05); Lowercase letters indicate differences between sites (CK and endosperm; P<0.05).

The study reported the presence of an endogenous inhibitor in the endosperm of seeds, which may also contribute to their dormancy (Shao et al., 2021). To investigate the presence of such an inhibitor in *S. paniculata* seeds’ endosperm, we compared the biological activity of different endosperm extracts with that of Chinese cabbage seeds by extracting and separating the endosperm extract. The findings revealed that each extract exhibited a distinct inhibitory effect on the germination rate, seedling height, and root length of Chinese cabbage (**Figure 4**). However, the degree of inhibition varied across different extraction phases, with all groups showing no significant differences except for the germination rate (**Figure 4A**), where notable variations were observed in both seedling height and root length. The average seedling height of Chinese cabbage under the methanol, petroleum ether, ethyl acetate, ether, and water phases were 6.82mm, 8.93mm, 10.39mm, 8.4mm, and 14.11mm respectively, representing proportions of 57.31%, 75.04%, 87.31%, 70.58%, and 118.57% compared to the control (11.90mm) (**Figure 4B**). The average root lengths of Chinese cabbage in the methanol phase, petroleum ether, ethyl acetate, ethyl ether phase, and water phase were 6.15 mm, 28.34 mm, 33.42 mm, 39.45 mm, and 51.70 mm respectively (Control: 51.78 mm), representing increases of 11.88%, 54.73%, 64.54%, 76.19%, and an astonishing improvement of almost a hundred percent (**Figure 4C**). In conclusion, methanol exhibited the most potent inhibitory effect on both seedling height and root length of Chinese cabbage (**Figures 4D–H**), thereby suggesting that the endogenous inhibitor of *S. paniculata* seeds predominantly resided within the methanol phase of the endosperm.

#### 3.4.2 Analysis of endocrine inhibitors of *S. paniculata* seed endosperm

**Figure 5:**
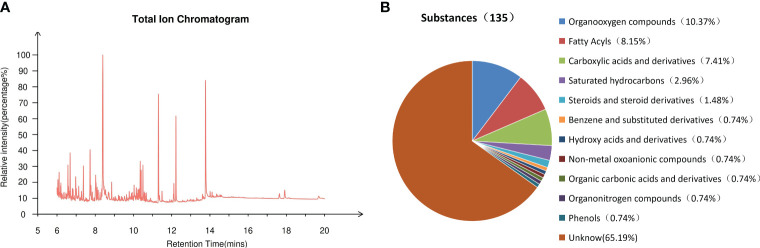
The investigation of germination inhibitors in the methanol phase of *S. paniculata* seed endosperm. **(A)** Total ion flow chromatogram; **(B)** Classification of 135 substances.

The methanol phase of endosperm, which exhibited the most potent inhibitory effect on Chinese cabbage seed growth, underwent further GC-MS qualitative analysis. **Figure 5A** illustrates the total ion current chromatograph of this methanol phase. After conducting an extensive search within the mass spectrometry system and cross-referencing with the standard spectrum, a grand total of 135 substances were successfully detected. These included a remarkable array of compounds, such as 14 exquisite organic oxygen compounds, 11 distinguished fatty acids, 10 refined carboxylic acids and their derivatives, 4 pristine saturated hydrocarbons, 2 elegant steroids and steroid derivatives, as well as one exceptional phenol alongside other captivating substances (**Figure 5B**). According to its peak area selected 20 compounds with high relative content (**Table 1**), the methanolic phase of the endosperm contains a plethora of fatty acids, phenolics, and organooxygen compounds. Notably, three fatty acid compounds were identified: hexadecanoic acid (11.19%), octadecanoic acid (8.73%), and (9Z)-octadecenoic acid (0.84%). Additionally, m-cresol was found as a representative phenolic species. Furthermore, phosphoric acid (1.86%) was detected as a nonmetal oxoanionic compound while dodecane (1.70%) represented a saturated hydrocarbon in this phase. Moreover, putrescine (1.42%) emerged as an organonitrogen compound alongside ten unidentified compounds.

**Table 1:** The composition and relative concentrations of compounds in methanol phase of *S. paniculata* endosperm.

Number	Time	Name	Super class	Class	Relative content(%)
1	11.309	Hexadecanoic acid	Lipids and lipid-like molecules	Fatty Acyls	11.19
2	13.774	alpha-D-Glucopyranoside, beta-D-fructofuranosy	Unknow	–	10.08
3	12.223	1-Ethylnaphthalene	Unknow	–	8.73
4	12.223	Octadecanoic acid	Lipids and lipid-like molecules	Fatty Acyls	8.73
5	7.730	m-Cresol	Benzenoids	Phenols	8.69
6	10.355	L-Sorbinose	Unknow	–	5.06
7	10.355	D-Fructose	Organic oxygen compounds	Organooxygen compounds	5.06
8	6.686	Phosphate	Unknow	–	4.17
9	6.686	Glycerol	Organic oxygen compounds	Organooxygen compounds	4.17
10	8.037	3-Amino-2-methylpropanoic acid	Unknow	–	2.77
11	10.408	D- (-) -Fructose	Unknow	–	2.73
12	10.497	alpha-D-Galactose	Organic oxygen compounds	Organooxygen compounds	1.99
13	6.689	Phosphoric acid	Homogeneous non-metal compounds	Non-metal oxoanionic compounds	1.86
14	8.540	Dodecane	Hydrocarbons	Saturated hydrocarbons	1.70
15	10.179	DL-Methionine sulfone	Unknow	–	1.49
16	7.282	Putrescine	Organic nitrogen compounds	Organonitrogen compounds	1.42
17	8.687	2-Naphthaldehyde	Unknow	–	1.07
18	12.116	9-Octadecenoic acid	Unknow	–	0.97
19	9.955	L-Methionine S-oxide	Unknow	–	0.89
20	12.121	(9Z)-Octadecenoic acid	Lipids and lipid-like molecules	Fatty Acyls	0.84

#### 3.4.3 Hexadecanoic acid, octadecanoic acid, and m-cresol exhibit significant inhibitory effects on the growth of Chinese cabbage seeds

**Figure 6:**
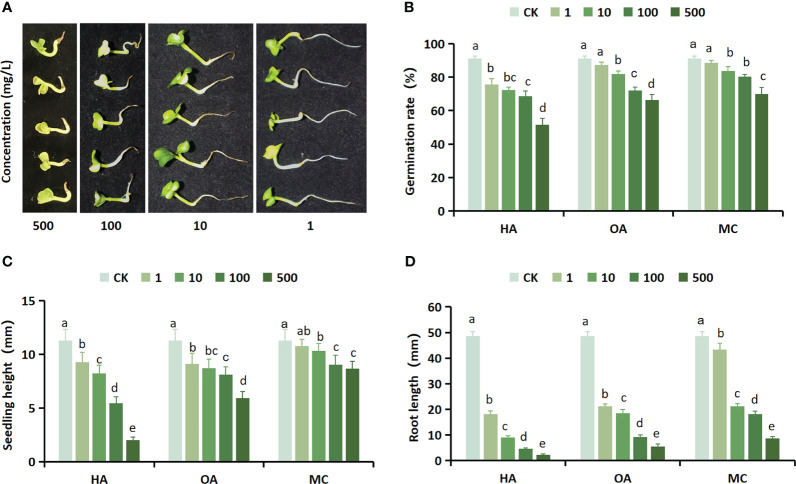
The growth of Chinese cabbage seeds is significantly inhibited by three potent inhibitors. **(A)** The effect of different concentrations of hexadecanoic acid on the growth of Chinese cabbage; **(B)** Effect of three inhibitors at different concentrations on the germination rate of Chinese cabbage seeds; **(C)** Effect of different concentrations of three inhibitors on Chinese cabbage seeds seedling heights; **(D)** Effects of three inhibitors at different concentrations on Chinese cabbage seeds root length. HA, hexadecanoic acid; OA, octadecanoic acid; MC, m-cresol.

The aim of this study is to investigate whether the application of GC-MS for detecting inhibitory substances has an inhibitory effect on seed germination. For this purpose, three inhibitors with higher content, namely hexadecanoic acid, octadecanoic acid, and m-cresol, were selected to conduct a germination experiment on Chinese cabbage seeds. The results revealed that the three inhibitors exhibited conspicuous inhibitory effects on the rate of seed germination, height of seedlings, and length of roots in Chinese cabbage. Moreover, these inhibitory effects became increasingly pronounced with higher concentrations of treatment (**Figures 6A–D**). The application of inhibitor treatment at a concentration of 500 mol/L resulted in a significant reduction in the germination rate of Chinese cabbage seeds, with hexadecanoic acid, octadecanoic acid, and m-cresol causing decreases of 39.66%, 25.00%, and 21.33% respectively. Furthermore, compared to the control group, seedling height decreased by 9.29mm, 5.37mm, and 2.64mm respectively; while root length showed reductions of 46.46mm, 43.10mm, and 39.89mm. The reduction degree of seed germination rate, seedling height, and root length of the three inhibitors compared with the control was synthesized. It can be concluded that all three inhibitors possess significant inhibitory effects on the growth of Chinese cabbage, with hexadecanoic acid exhibiting the strongest inhibition followed by octadecanoic acid and m-cresol in decreasing order.

## 4 Discussion

The exterior morphology of certain seeds may exhibit signs of maturity, accompanied by mature seed traits; however, their interior physiological and biochemical processes necessitate a specific stage of development for completion (Baskin and Baskin, 2021). The temperature must be elevated at the beginning of stratification to stimulate embryo growth and differentiation, while it should be reduced towards the end of stratification to facilitate the physiological and biochemical processes of the seed embryo (Han et al., 2022). The morphological structure of the *S. paniculata* seed embryo was fully developed prior to seed maturity, exhibiting a complete and intricate embryonic architecture. The dormancy observed in this experiment cannot be attributed to any morphological aftermath of the embryo.

The failure of germination in seeds with high vitality and well-developed embryos under suitable conditions can be attributed to either the impermeability of the seed coat or hard endocarp, which acts as a mechanical restriction. The permeability of these structures plays a crucial role in determining successful germination by facilitating water absorption (Manz et al., 2005; Farhana and Conor, 2013; Gao et al., 2022). In our study, the lignification of *S. paniculata* endocarp was observed to be severe, and water uptake in cracked seeds was significantly higher compared to intact seeds, thereby indicating that seed endocarp lignification causes impermeability and potentially contributes to the mechanical dormancy of *S. paniculata* seeds. However, Baskin et al. have revealed that the lack of water uptake cannot be solely ascertained by comparing imbibition in scarified versus non-scarified seeds. They also consider mechanical dormancy as an integral part of physiological dormancy (Baskin and Baskin, 2004). This is because the mechanical restriction imposed by the endocarp barrier, which acts as a covering layer or layers, hinders embryo growth and makes it difficult for the radicle to penetrate during germination, thereby resulting in low growth potential of the embryo in intact dormant seeds (Chen et al., 2021; Jang et al., 2023).

The inhibition of seed material, specifically physiological dormancy, may also contribute to the dormancy of *S. paniculata* seeds. The present study investigates the primary types of inhibitors responsible for inducing physiological dormancy. By comparing the results of bioassay for each organic phase, it was discovered that methanol derived from the seed embryo exerts a significant inhibitory impact on Chinese cabbage growth. Therefore, the substances identified in the methanol phase of the endosperm may be intricately linked to its physiological dormancy. Benzoic acid, palmitic acid, and stearic acid are crucial endogenous inhibitors of seed dormancy in *Prunus sargen*tii (Song et al., 2021), While phenol, catechol, and 2,6-di-tert-butylp-cresol serve as significant endogenous inhibitors of seed testa in *Paeonia ostii* ‘Fengdan’, organic acids such as benzoic acid, oleic acid, palmitic acid, and stearic acid emerge as the primary inhibitory substances of its endosperm (Li Y. et al., 2022). These organic acids and phenolic substances primarily impede seed germination by obstructing the process of seed water absorption, suppressing respiration, inhibiting the activity of biological enzymes, and restraining radicle growth (Li et al., 2013; Grainge et al., 2022; Zarban et al., 2022). In recent years, the isolation and identification of germination-inhibitory compounds have emerged as a pivotal aspect in researching seed dormancy mechanisms (Zhou et al., 2023).

The GC-MS test results reveal significant inhibition of Chinese cabbage growth in the methanol phase endosperm, with a remarkably high relative content of hexadecanoic acid at 11.19%. Additionally, it contains a certain amount of octadecanoic acid and m-cresol. These findings resemble the endogenous inhibitory species found in *Prunus sargentii* and *Paeonia ostii* ‘Fengdan’ seeds. The presence of these fatty acids and phenols may serve as a pivotal factor contributing to the profound inhibitory impact on the growth of Chinese cabbage. Hence, it can be inferred that the fatty acid and phenolic compounds are likely associated with seed dormancy. The verification process involved the selection of three inhibitors, namely hexadecanoic acid, octadecanoic acid, and m-cresol. It was found that these inhibitors exhibited significant inhibitory effects on the growth of Chinese cabbage, which aligns with previous research findings on the endogenous inhibitor *Paeonia ostii* ‘Fengdan’ (Rubio et al., 2017). These results provide a solid theoretical foundation for further exploration into seed germination in *S. Paniculata*.

## 5 Conclusion

In this study, we utilized *S. paniculata* seeds as experimental materials and conducted an investigation into the types and causes of seed dormancy in *S. paniculata* by analyzing the morphological and developmental characteristics of its seed embryo, exploring the water permeability property of the endocarp, and examining the presence of endogenous inhibitors. The embryo of *S. paniculata* is exquisitely formed, boasting a complete embryonic structure without any signs of morphological dormancy. Throughout the course of seed development, the endocarp undergoes a process of extensive lignification, culminating in near-complete lignification at maturity. As a result, the seeds exhibit low water permeability and mechanical restriction. Furthermore, the presence of endogenous inhibitors is responsible for the physiological dormancy of *S. paniculata* seeds, and its endosperm extract exerts a significant inhibitory effect on the growth of Chinese cabbage. Preliminary GC-MS analysis unveiled that the types of endogenous inhibitors primarily encompass organic acids and phenolics, such as hexadecanoic acid, octadecanoic acid, and m-cresol. The further validation study demonstrated the significant inhibitory effects of hexadecanoic acid, octadecanoic acid, and m-cresol on the growth of Chinese cabbage. Our findings suggest that seeds of *S. paniculata* possess inherent limitations in water absorption and contain endogenous inhibitors, both of which contribute to physiological dormancy. These findings indicate potential strategies for breaking seed dormancy by weakening the protective layer of the embryo covering (endocarp) and reducing the content of endogenous inhibitors in the endosperm.

## Data availability statement

The original contributions presented in the study are included in the article/supplementary material. Further inquiries can be directed to the corresponding authors.

## Author contributions

QT: Data curation, Software, Writing – original draft. YC: Investigation, Writing – original draft. LJ: Data curation, Writing – original draft. JC: Data curation, Writing – original draft. CL: Writing – original draft. WZ: Writing – original draft. PL: Writing – original draft. QL: Writing – original draft.
